# Poor diet quality is associated with self-reported knee pain in community-dwelling women aged 50 years and older

**DOI:** 10.1371/journal.pone.0245630

**Published:** 2021-02-16

**Authors:** Woo-young Shin, Jung-ha Kim

**Affiliations:** Department of Family Medicine, Chung-Ang University Medical Center, Chung-Ang University College of Medicine, Seoul, Republic of Korea; Emory University School of Medicine, UNITED STATES

## Abstract

In the current study, we aimed to examine the association between knee pain and diet quality in women aged ≥ 50 years using data from the Korea National Health and Nutrition Examination Survey. This was a population-based, cross-sectional study. Diet quality was assessed using the Diet Quality Index-International (DQI-I), and knee pain and osteoarthritis were self-reported. A multivariate logistic regression model was used to adjust for age, body mass index, household income, marital status, education, occupation, smoking status, hazardous alcohol use, regular physical activity, menopause, and chronic diseases, including hypertension, diabetes, dyslipidemia, osteoarthritis, and depression. A total of 3,881 women were included in this study, and the prevalence of knee pain was 25.4%. The intakes of total energy, protein, and fat were lower in women with knee pain than in those without (all P < 0.01), while the carbohydrate intake was higher (P = 0.01). No significant differences were noted in the scores for variety, overall balance, and moderation components, except for the item of total fat intake, between the DQI-I scores for women with and without knee pain, after adjusting for age. Women without knee pain showed higher scores in several items of the adequacy component (P < 0.05) than did women with knee pain. The total DQI-I scores were lower in women with knee pain than in women without knee pain, after adjusting for covariates, including osteoarthritis (OR = 0.985, 95% CI = 0.973–0.997, P = 0.01). Knee pain independent of osteoarthritis was associated with poor diet quality in community-dwelling women aged ≥ 50 years.

## Introduction

Knee pain is a common musculoskeletal symptom in adults aged 50 years and older [1]. The prevalence of knee pain has increased at a remarkable rate in the past several decades and is frequently caused by osteoarthritis [2]. Demographic, social, and psychological factors, as well as the structural destruction of the joints are related to knee pain, as with other forms of musculoskeletal pain [3,4]. Bartley et al. suggested that women exhibited greater sensitivity to multiple pain modalities compared to men, among adults with symptomatic knee osteoarthritis [5]. Another study demonstrated that the prevalence of knee pain was higher in elderly women than in elderly men and was also shown to increase with age [6]. Knee pain is known to be one of the major factors contributing to total knee arthroplasty among knee osteoarthritis patients [2]. Individuals with knee pain are reported to experience a lower quality of life than those without knee pain [7]. Furthermore, the quality of life is poorer in patients with knee pain than in patients diagnosed with radiographic knee osteoarthritis who do not experience knee pain, independently of osteoarthritis [8].

An association between musculoskeletal disorders, such as sarcopenia, or osteoporosis and malnutrition has been previously reported [9,10]. Dietary fiber, omega-3 polyunsaturated fatty acid, and consumption of strawberries are all associated with knee osteoarthritis or joint pain [11–13]. Recently, comprehensive approaches such as analysis of diet quality have proven to be more useful than the analysis of the role of single nutrients or foods when attempting to explain the relationship between diet and disease [14].

The current suggested definition of a high-quality diet is “a diversified, balanced, and healthy diet, which provides energy and all essential nutrients for growth and a healthy and active life.” [15]. Indices of overall dietary quality, such as the healthy eating index, Mediterranean diet score, overall nutritional quality index, and the dietary approaches to stop hypertension (DASH) score, have been used as nutritional indicators [16–19]. These measures are commonly used to assess dietary impact on the quality of life, frailty, chronic diseases, and mortality [16–19]. However, no previous study has investigated the relationship between diet quality and knee pain, especially among older women, defined as a vulnerable group with a greater risk of increased pain, relative to men or younger adults. The aim of the current study was to identify whether there is an association between knee pain and diet quality using the diet quality index-international (DQI-I), which is a useful tool for dietary assessment. This study was performed in community-dwelling older Korean women.

## Materials and methods

### Study participants

This study was based on data acquired from the Korea National Health and Nutrition Examination Survey (KNHANES) 2013–2015. The KNHANES was conducted by the Korea Centers for Disease Control and Prevention (KCDC) using a multistage clustered and stratified randomized sampling method based on the National Census data. All participants provided written informed consent. The KNHANES was approved by the Institutional Review Board of the KCDC (2013-07CON-03-4C, 2013-12EXP-03-5C, and 2015-01-02-6C).

**Fig 1:**
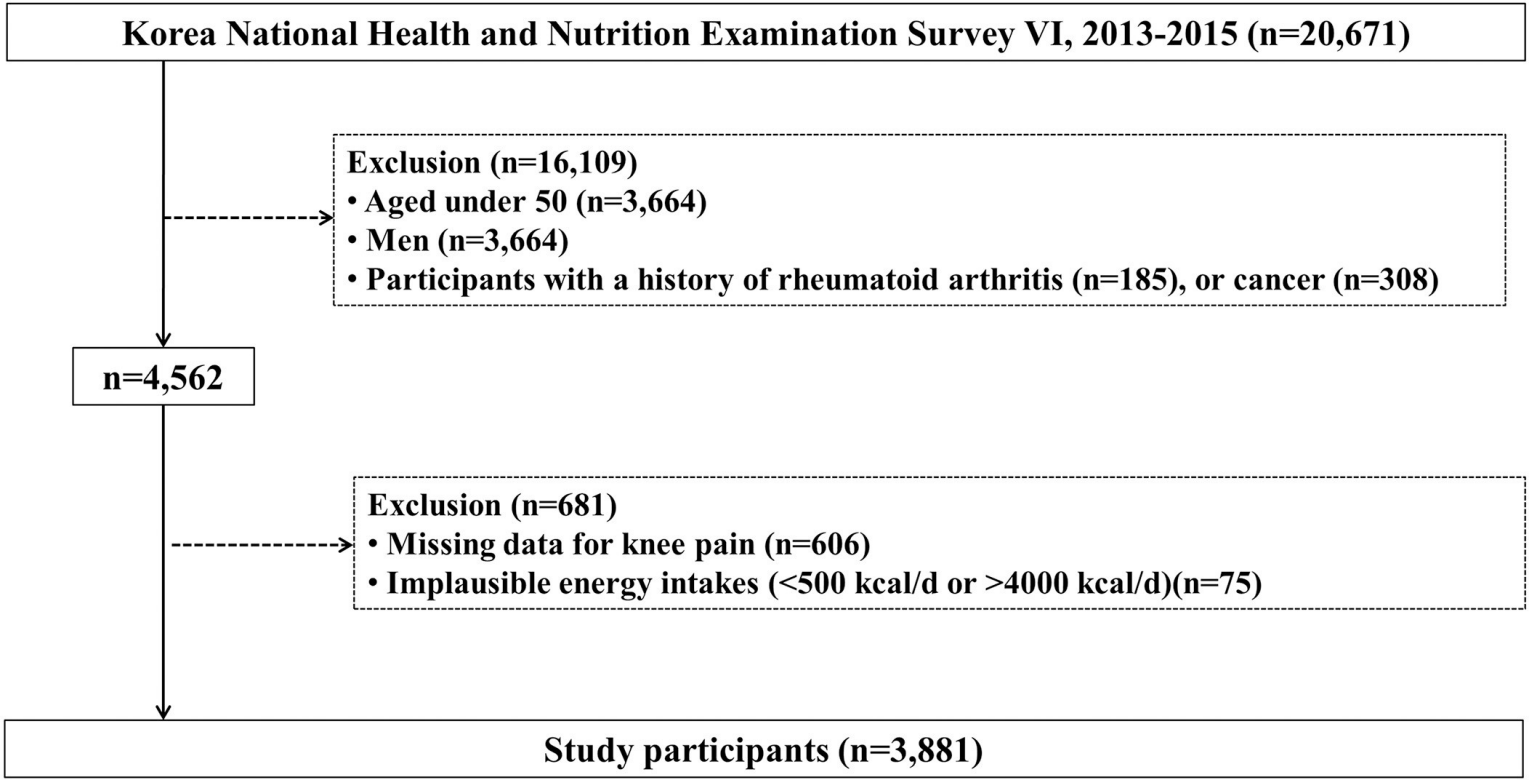
Study participants: Data from the KNHANES-VI (2013–2015).

Initially, a total of 20,671 candidates completed a health interview/examination and a nutritional survey. The following participants were excluded: 11,952 individuals aged under 50, 3,664 men, 493 people with a history of rheumatoid arthritis (n = 185) or cancer (n = 308), 606 participants who had missing data for knee pain, and 75 individuals with implausible energy intakes (< 500 kcal or > 4,000 kcal/d). This left a final analytic total of 3,881 Korean women (Fig 1).

### Dietary assessment

Dietary intake was self-reported by participants, using a 1-day, 24-hour dietary recall method. Two dietitians who were thoroughly acquainted with the protocols and techniques involved in the study conducted a computer-assisted personal interview in the participants’ homes. For those with a standard dietary intake, the interview was carried out 1 week after the participants had completed a health interview and physical examination. Data concerning all the foods and beverages consumed by the participants during the previous day were collected [20]. Total energy intake and dietary nutrient compositions and quantities were estimated using a standard Korean food composition table [21].

Diet quality was evaluated based on the DQI-I [22]. The index was calculated as a sum of the score from four categories that generate a measure of overall diet quality: variety, adequacy, moderation, and overall balance. The variety component includes the overall variety of food groups consumed (meat/poultry/fish/eggs, dairy/beans, grains, fruit, and vegetables) and within-group variety for the protein source (meat, poultry, fish, dairy, beans, and eggs). The second category assesses the adequacy of the intake of vegetables, fruits, grains, fiber, protein, iron, calcium, and vitamin C. We modified the scoring criteria for this category according to the recommended servings for Koreans, based on the total energy intake (in the case of vegetables, fruit, and grains), the adequate intake (in the case of fiber), or the recommended nutrient intake (in the case of iron, calcium, and vitamin C) [23]. The moderation category covers total fat, sodium, empty calorie foods, saturated fat, and cholesterol. We excluded empty calorie foods because they could not be estimated using our food composition table. The last category was overall balance, in which the ratios of macronutrients and fatty acids were included. In the current study, the total scores of the DQI-I ranged from 0 to 94 (S1 Table).

### Survey of knee pain and osteoarthritis

A simple dichotomous question (yes/no) was used for a self-reported measure of knee pain: “Have you experienced pain in your knee for over 30 days during the past 3 months?” Osteoarthritis was defined when the participants self-reported that they had been diagnosed with the disease by a medical doctor.

### Characteristics of the participants as covariates

The demographic variables collected for this study included age, household income, marital status, educational level, and occupation. Equalized household incomes were calculated as the total monthly household income divided by the square root of the total number of household members. A married person was defined as having married status and cohabiting. Higher education was defined as the participant having at least a college education. Occupations were classified into four groups as follows: non-manual workers (general managers, professionals, and office workers), service or sales workers, manual workers (skilled agricultural, forestry and fishery workers, craft and related trade workers, plant and machine operators or assemblers, and elementary workers), and unemployed (including homemakers and students). Information on physical activity was obtained using self-reported questionnaires. Regular physical activity was defined as high intensity activity (e.g., running or mountaineering) for at least 75 minutes during the week, moderately intense activity (e.g., light swimming, badminton, or walking) for at least 150 minutes during the week, or an equivalent combination of moderate- and high-intensity activities [24]. Participants who smoked at the time of the survey or who had smoked more than 100 cigarettes during their lifetime were considered current smokers. Hazardous alcohol use was defined as individuals with an alcohol use disorders identification test (AUDIT) score of 12 or more [25]. Menopausal status and chronic diseases diagnosed by a medical doctor were self-reported.

Body mass index (BMI) was calculated as body weight in kilograms divided by the height in meters squared (kg/m^2^). Body weight and height were measured while subjects were dressed in light clothing without shoes.

### Statistical analyses

All statistical analyses were performed using SAS 9.2 (SAS Institute Inc., Cary, NC, USA). Data are presented as the mean ± standard error (SE) for continuous variables and as a weighted percentage (SE) for categorical variables. A weighted t-test or weighted χ^2^-test was applied to compare characteristics between women with and without knee pain. A weighted analysis of covariance (ANCOVA) was used to examine the differences in the intakes of macronutrients, and the total and individual DQI-I score between the two groups was adjusted for age. The total DQI-I scores were compared between women with and without osteoarthritis, adjusting for BMI and age using a weighted ANCOVA, within each group (women with and without knee pain), respectively. Multivariate logistic regression analyses were used to evaluate the relationships between knee pain and the total DQI-I scores after adjusting for all confounders, including age, BMI, household income, marital status, educational level, total energy intake, occupation, smoking status, hazardous alcohol use, regular physical activity, menopause, and chronic diseases including hypertension, diabetes, dyslipidemia, osteoarthritis, and depression. For these analyses, we used a 5% significance level and survey procedures for complex sample designs.

## Results

### Baseline characteristics

A total of 3881 women with complete data were included in this study (Fig 1). The overall prevalence of knee pain was 25.4%, and 28.3% of the participants had self-reported doctor-diagnosed osteoarthritis. The baseline characteristics of the study population are shown in Table 1. Current smoking (P = 0.78) and hazardous alcohol use (P = 0.22) were not significantly different between women with and without knee pain. All the other variables differed between the two groups.

**Table 1:** Baseline characteristics of women with and without knee pain.

	Total participants (n = 3,881)	Women without knee pain (n = 2,824)	Women with knee pain (n = 1,057)	P-value^a^
Percentage		74.6 (0.8)	25.4 (0.8)	
Age (years)	62.6 ± 0.2	61.5 ± 0.2	66.1 ± 0.3	< 0.01
Household income				< 0.01
Quartile 1	28.7 (1.0)	23.9 (1.0)	42.9 (1.9)	
Quartile 2	25.7 (0.9)	26.2 (1.0)	24.3 (1.5)	
Quartile 3	21.5 (0.8)	22.7 (1.0)	17.9 (1.5)	
Quartile 4	24.1 (1.0)	27.3 (1.2)	14.8 (1.3)	
Higher education: at least college	10.9 (0.7)	12.7 (0.9)	5.6 (0.9)	< 0.01
Occupation				< 0.01
Non-manual worker	6.1 (0.5)	7.1 (0.6)	3.2 (0.6)	
Service and sales worker	14.5 (0.7)	16.4 (0.8)	9.0 (1.1)	
Manual worker	22.0 (0.9)	21.3 (1.0)	24.2 (1.6)	
None	57.4 (1.1)	55.2 (1.2)	63.6 (1.8)	
Married	69.4 (1.0)	72.6 (1.1)	59.8 (1.8)	< 0.01
Body mass index (kg/m^2^)	24.3 ± 0.1	24.0 ± 0.1	24.9 ± 0.1	< 0.01
Current smoker	2.8 (0.3)	2.8 (0.4)	2.6 (0.6)	0.78
Hazardous alcohol use^b^	1.6 (0.2)	1.7 (0.3)	1.1 (0.4)	0.22
Regular physical activity	32.1 (1.1)	33.8 (1.3)	27.0 (1.7)	< 0.01
Menopause	89.5 (0.6)	88.4 (0.7)	92.9 (0.9)	< 0.01
Self-reported chronic disease				
Hypertension	37.5 (0.9)	34.8 (1.1)	45.3 (1.8)	< 0.01
Diabetes	12.9 (0.7)	11.7 (0.7)	16.4 (1.4)	< 0.01
Dyslipidemia	26.7 (0.8)	25.1 (1.0)	31.6 (1.6)	< 0.01
Osteoarthritis	28.3 (0.8)	18.0 (0.8)	58.4 (1.9)	< 0.01
Depression	8.0 (0.5)	6.8 (0.6)	11.3 (1.1)	< 0.01

Data are expressed as mean ± standard error or percentage (standard error).

^a^Analyzed using a weighted t-test or weighted χ^2^-test to assess differences between women with and without knee pain. Analyses performed using PROC SURVEYREG and PROC SURVEYFREQ, respectively.

^b^Alcohol use disorder identification test (AUDIT) score ≥ 12.

### Assessment of macronutrient intakes and dietary quality using the diet quality index-international

**Fig 2:**
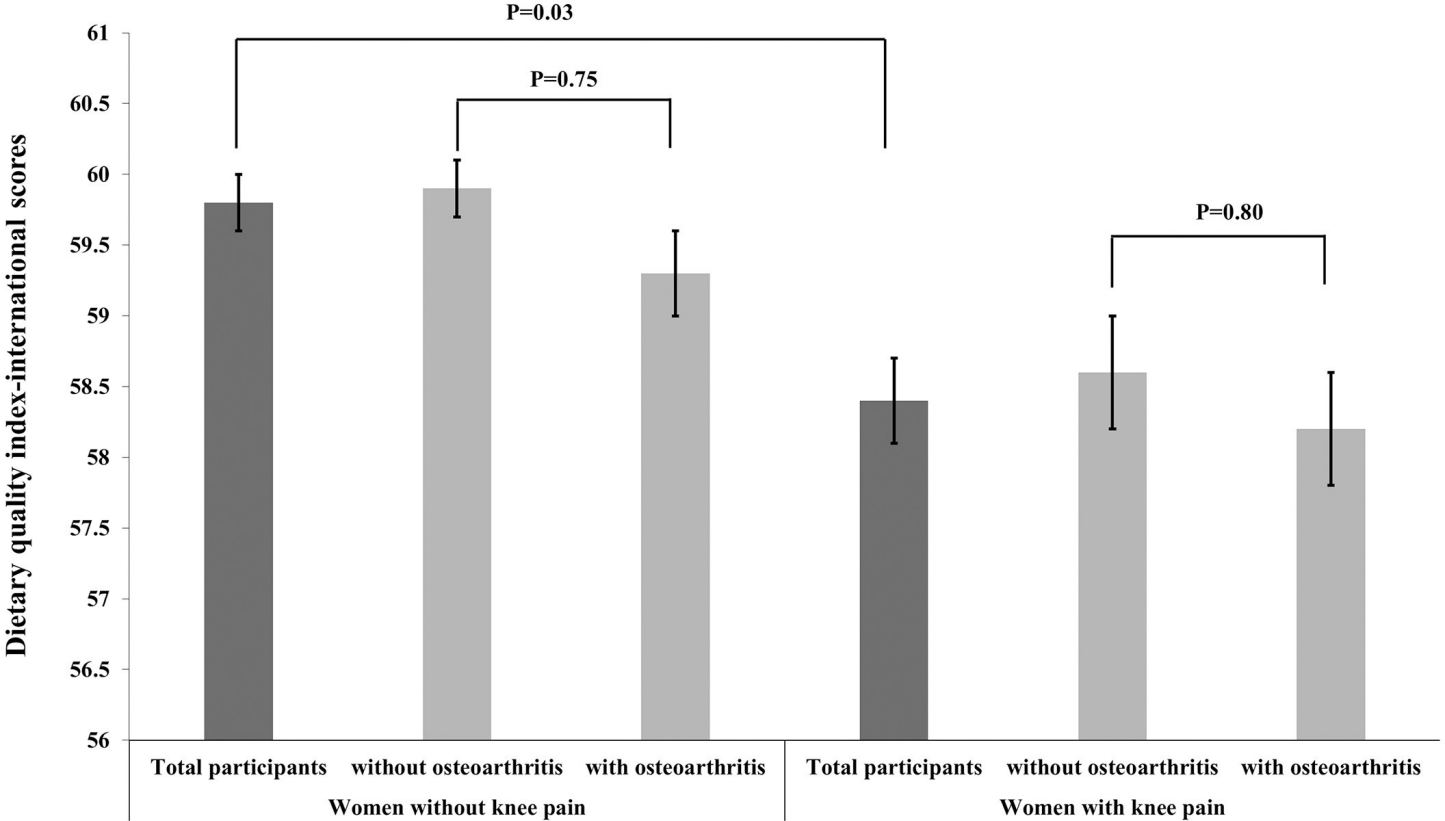
Comparison of the dietary quality index-international (DQI-I) scores using a weighted ANCOVA, adjusting for age and body mass index. There were no significant differences in the DQI-I scores between women with and without osteoarthritis within the two groups (women with and without knee pain), respectively. The DQI-I scores in women without knee pain were higher than the scores in women with knee pain.

Intakes of total energy, protein, and fat were lower (all P < 0.01) and carbohydrate intake was higher (P = 0.01) in women with knee pain than in those without (Table 2). The total DQI-I scores ranged from 18.5 to 85.8. The total scores in women without knee pain were higher than those in women with knee pain after adjusting for age (P < 0.01). There were no significant differences in the total DQI-I scores of women without osteoarthritis (59.9 ± 0.2) and women with osteoarthritis (59.3 ± 0.3), or between women without knee pain (P = 0.75), and between women without osteoarthritis (58.6 ± 0.3) and women with osteoarthritis (58.2 ± 0.4) among participants with knee pain (P = 0.80) after adjusting for age and BMI (Fig 2). Table 2 shows that the scores of most items in the adequacy component of the survey, apart from vegetables and grains, are higher and the total fat intake score in the moderation component is lower among women without knee pain than among women with knee pain.

**Table 2:** Macronutrient intakes and dietary quality index-international (DQI-I) scores for women with and without knee pain.

	Total participants (n = 3,881)	Women without knee pain (n = 2,824)	Women with knee pain (n = 1,057)	P-value^a^
Macronutrient intakes				
Total energy (kcal/d)	1,652.9 ± 12.8	1,687.6 ± 14.3	1,551.4 ± 22.2	< 0.01
Protein (% of total energy intake)	13.1 ± 0.1	13.3 ± 0.1	12.7 ± 0.1	< 0.01
Carbohydrate (% of total energy intake)	70.7 ± 0.2	70.0 ± 0.3	72.8 ± 0.4	0.01
Fat (%) of total energy intake	14.9 ± 0.2	15.5 ± 0.2	13.1 ± 0.3	< 0.01
DQI-I scores				
Total DQI-I score	59.5 ± 0.2	59.8 ± 0.2	58.4 ± 0.3	< 0.01
Variety				
Overall food group variety	7.2 ± 0.1	7.4 ± 0.1	6.9 ± 0.1	0.06
Within-group variety for protein source	1.0 ± 2.4E^-2^	1.0 ± 2.6E^-2^	0.9 ± 3.9E^-2^	0.11
Adequacy				
Vegetable group^b^^,^ ^c^	4.8 ± 1.3E^-2^	4.9 ± 1.3E^-2^	4.8 ± 2.7E^-2^	0.21
Fruit group^c^^,^ ^d^	3.4 ± 4.7E^-2^	3.5 ± 0.1	3.2 ± 0.1	0.04
Grain group^c^^,^ ^e^	2.0 ± 1.7E^-2^	2.0 ± 1.9E^-2^	2.0 ± 2.9E^-2^	0.46
Fiber^c,^ ^f^	4.3 ± 2.2E^-2^	4.4 ± 2.4E^-2^	4.1 ± 4.6E^-2^	< 0.01
Protein^c^	4.9 ± 5.9E^-3^	4.9 ± 6.3E^-3^	4.9 ± 1.1E^-2^	0.03
Iron^c^^,^ ^g^	4.8 ± 9.1E^-3^	4.9 ± 8.9E^-3^	4.8 ± 2.1E^-2^	< 0.01
Calcium^c^^,^ ^g^	2.6 ± 2.7E^-2^	2.7 ± 2.9E^-2^	2.4 ± 0.1	0.02
Vitamin C^c^^,^ ^g^	3.3 ± 3.9E^-2^	3.4 ± 4.2E^-2^	3.0 ± 0.1	< 0.01
Moderation				
Total fat	5.1 ± 3.4E^-2^	5.0 ± 4.1E^-2^	5.4 ± 0.1	< 0.05
Sodium	3.4 ± 0.1	3.3 ± 0.1	3.7 ± 0.1	0.08
Saturated fatty acids	5.5 ± 2.6E^-2^	5.5 ± 3.2E^-2^	5.6 ± 4.6E^-2^	0.23
Cholesterol	5.2 ± 4.0E^-2^	5.2 ± 4.6E^-2^	5.4 ± 0.1	0.39
Overall balance				
Macronutrient ratio^h^ (carbohydrate:protein:fat)	0.9 ± 3.0E^-2^	0.9 ± 3.6E^-2^	0.7 ± 0.1	0.27
Fatty acids ratio	0.9 ± 2.4E^-2^	1.0 ± 3.0E^-2^	0.8 ± 4.5E^-2^	0.09

Data are expressed as mean ± standard error.

^a^Analyzed using a weighted analysis of covariance (ANCOVA) adjusting for age between women and without knee pain.

^b^Including vegetables, mushrooms, and seaweeds: ≥ 5 servings/d = 5 was defined as an energy intake < 1,300 kcal; ≥ 6 servings/d = 5 was defined as an energy intake of between ≥ 1,300 and < 1,800 kcal; ≥ 7 servings/d = 5 was defined as an energy intake of between ≥ 1,800 and < 1,900 kcal; ≥ 8 servings/d = 5 was defined as an energy intake of between ≥ 1,900 and < 2,600 kcal; and ≥ 9 servings/d = 5 was defined as an energy intake of ≥ 2,600 kcal.

^c^ Used as a continuous variable.

^d^≥ 1 servings/d = 5 was defined as an energy intake of <1,800 kcal; ≥ 2 servings/d = 5 was defined as an energy intake of between ≥ 1,800 and less than 2,400 kcal; and ≥ 4 servings/d = 5 was defined as an energy intake of ≥ 2,400 kcal.

^e^ Including cereals, potatoes, and starches. ≥ 1.5 servings/d = 5, was defined as an energy intake of <1,200 kcal; ≥ 2 servings/d = 5 was defined as an energy intake of between ≥ 1,200 and less than 1,400 kcal; ≥ 2.5 servings/d = 5 was defined as an energy intake of between ≥ 1,400 and less than 1,600 kcal; ≥ 3 servings/d = 5 was defined as an energy intake of between ≥1,600 and less than 2,000 kcal; ≥ 3.5 servings/d = 5 was defined as an energy intake of between ≥ 2,000 and less than 2,300 kcal; and ≥ 4 servings/d = 5 was defined as an energy intake ≥ 2,300 kcal.

^f^ Scoring system based on the adequate intake value for the Republic of Korea.

^g^ Scoring system based on the recommended nutrient intake value for the Republic of Korea.

^h^ Ratio of energy from carbohydrate to protein to fat.

### Associations between knee pain and dietary quality were assessed using the diet quality index-international

Table 3 shows the independent association between knee pain and the total DQI-I scores (OR = 0.985, 95% CI = 0.973–0.997, P = 0.01).

**Table 3:** Multivariate logistic regression analysis to identify independent clinical variables associated with knee pain.

	Odds ratio	95% Confidence interval	P-value^a^
Age (years)	1.014	1.001–1.028	0.04
Household income: Q1 vs. Q4	1.703	1.260–2.302	< 0.01
Higher education: college or more	0.611	0.381–0.979	0.04
Osteoarthritis: yes vs. no	5.306	4.376–6.434	< 0.01
Depression: yes vs. no	1.435	1.080–1.906	0.01
Dietary quality index-international scores	0.985	0.973–0.997	0.01

^a^Adjusted for all confounders, including age; body mass index; household income; marital status; educational level; occupation; smoking status; hazardous alcohol use; regular physical activity; menopause; chronic diseases, including hypertension, diabetes, dyslipidemia, osteoarthritis, and depression; and total energy intake.

## Discussion

In this cross-sectional study, knee pain was associated with a low DQI-I score, and the overall prevalence of knee pain in women was 25.4%. Women without knee pain had higher scores than women with knee pain in the adequacy component of the survey. The DQI-I scores did not differ significantly between women with and those without osteoarthritis, irrespective of whether or not they experienced knee pain.

Poor diet quality and malnutrition in older adults are important areas of concern. Older adults are particularly vulnerable to malnutrition because aging may cause or worsen physical and psychological diseases, cognitive impairment, and dental problems [26]. Older adults may also experience socioeconomic changes, and increased age can influence nutritional status. Furthermore, malnutrition has been reported to contribute to a decline in general health, reduced physical and cognitive functional status, delay in recovery from illness, and increased risk of mortality in older persons [27]. Indeed, it is estimated that 1 in 6 members of the older population in Korea are malnourished or at risk of malnutrition [28]. With a predicted global increase in life expectancy, approximately 24.3% of the population in Korea is expected to be over 65 years by 2030, and the population aged 80 years or older will grow from 11.5% to 21.0% worldwide [29]. This will result in an increase in elderly individuals at a risk of poor diet quality.

We assessed the participants’ dietary intake using the 24-hour recall method in this study. The 24-hour recall method is the most widely used in large-scale nutrition epidemiology and diet-health outcomes association studies, such as national health and nutritional surveys in several countries, as a valid reliable assessment tool [30–32]. In this study, the use of the 24-hour recall was administered by well-trained interviewers at each participant’s home for a direct face to face interview to facilitate the quantification of household measures, which might reduce possible errors and bias among the participants [32]. The information on dietary intake was collected via an open questionnaire with elaborate interview support materials, such as examples of dishes and photographs of various serving sizes, and the possible recall bias to obtain reliable data on food intake could be minimized when estimating the portion size. It could help produce a comprehensive and detailed description of all foods and beverages consumed and identify the intake of energy and nutrients.

Poor diet quality may be associated with increased susceptibility to pain, and poor diet quality can induce thermal and mechanical hypersensitivity, as well as persistent pain following inflammatory provocation [33]. The standard American diet, which is regarded as a poor diet, has been shown to cause an elevation of microglial activation in the spinal cord, and it might contribute to increased pain and systemic inflammation [34,35]. Mattei et al. reported that poor diet quality is associated with an increase in high sensitivity C-reactive protein (hs-CRP), which is an index of low-grade systemic inflammation [36]. Among older adults with knee pain, elevated circulating levels of hs-CRP and pro-inflammatory cytokines, such as interleukin (IL)-6 and tumor necrosis factor-alpha (TNF-α), have been also found in other previous studies [37], which may result in the prolonged sensitization and hypersensitivity of C fibers transmitting pain signals from the knee joint to the spinal cord [38,39]. Meanwhile, recent reports have demonstrated that the association between obesity and body pain can be modulated by the consumption of food with anti-inflammatory effects [40]. We found that the participants’ intake of micronutrients increased in parallel with the increasing quartiles of the DQI-I score in our study (S2 Table). Low DQI-I scores represent a lowered intake of micronutrients, including riboflavin and zinc [22].

We found that low-fat or high-carbohydrate intakes were associated with knee pain, and this result is in keeping with previous evidences to suggest that a ketogenic diet, a high-fat and low-carbohydrate diet, may alleviate pain [41,42]. In addition, our results showed that energy intake was lower in women with knee pain than in women without, despite a high BMI. This could be because obese women with knee pain tried to lose weight for pain relief. Otherwise, another possible explanation for the lower intake of energy observed in those with knee pain might be attributed to possible depression or decrease in physical activity, caused by pain, that lead to a loss of appetite among individuals with knee pain. Indeed, the group with knee pain included less individuals who conducted regular physical activity than the group without knee pain in our study.

Knee pain is associated with old age, female sex, obesity, smoking, low educational level, manual occupation or knee-straining work, previous knee injuries, and radiographic osteoarthritis [7,43]. There have been limited studies on the dietary or nutritional risk factors of knee pain, although dietary risk factors for knee osteoarthritis have been reported [44,45]. Despite the strong association between knee pain and osteoarthritis, we did not find that self-reported osteoarthritis modified the association between knee pain and diet quality. This result was unexpected, and knee pain may be a systemic and underlying health problem as well as simply an isolated symptom caused by structural damage in relation to the mechanical loading of the knee joint. While, there have been evidences that individuals with poorer diet quality are more common in those with lower socioeconomic status including educational level and household income [46,47]. We calculated Variance Inflation Factor (VIF) between DQI-I and either the educational level or the household income among the participants, and all the values (less than 1.5) indicated no significant multicollinearity in our study. The educational level and the household income could be included as independent variables. The association between diet quality and knee pain showed that was persistent even after adjusting for these socioeconomic factors.

There are some limitations to this study. First, this was a cross-sectional study, which made it difficult to clarify the causality between diet quality and knee pain. For example, it is possible that knee pain led patients to change their dietary intake to relieve pain, as previously mentioned. Second, the use of a 1-day, 24-hour dietary recall method may not be appropriate for the assessment of diet quality. To adequately determine an individual’s habitual diet, repeated 24-hour dietary recalls have been recommended [48]. Furthermore, depending on the recall of the participants, under- or over-reporting could have occurred in this study. Third, both knee pain and osteoarthritis were self-reported in this study, and the status of radiographic criteria, such as joint space narrowing or osteophytes, for the participants could not be assessed. Finally, no blood markers of inflammation were measured. Therefore, we did not directly reveal whether the relationship between knee pain and diet quality was mediated by systemic inflammation.

## Conclusions

In conclusion, we found that knee pain was associated with poor diet quality, independently on osteoarthritis. This finding indicates that an adequate high-quality diet, ensured by providing dietary education or counseling, are required to relieve knee joint pain, as well as weight management correcting imbalances between caloric intake and expenditure. Future prospective studies need to directly examine how diet quality influences the development of knee pain in individuals with or without obesity and the underlying mechanism linking knee pain and diet quality.

## Supporting information

S1 Table Categories and scoring of the dietary quality index-international (DQI-I). (DOCX)

S2 Table Intake of micronutrients according to the DQI-I score quartiles. (DOCX)
