# Supplementary material for: Poor diet quality is associated with self-reported knee pain in community-dwelling women aged 50 years and older
Source: PLoS One. 2021 Feb 16;16(2):e0245630. doi: 10.1371/journal.pone.0245630 (PMC7886155; doi:10.1371/journal.pone.0245630)
Supplement: S1 Table — (DOCX) [file pone.0245630.s001.docx]

**S1 Table.** **Categories and scoring of the dietary quality index-international (DQI-I)**

| Category | Score (points) | Scoring criteria |
| --- | --- | --- |
| Total DQI-I score: | 0–94 |  |
| Variety | 0–20 |  |
| Overall food group variety  (meat/poultry/fish/eggs; dairy/beans; grains; fruit; vegetable) | 0–15 | ≥ 1 serving from each food group/d = 15 |
|  |  | Any 1 food group missing/d = 12 |
|  |  | Any 2 food groups missing/d = 9 |
|  |  | Any 3 food groups missing/d = 6 |
|  |  | ≥ 4 food groups missing/d = 3 |
|  |  | None from any food groups = 0 |
| Within-group variety for protein source (meat, poultry, fish, dairy, beans, eggs) | 0–5 | ≥ 3 different sources/d = 5 |
|  |  | Two different sources/d = 3 |
|  |  | From one source/d = 1 |
|  |  | None = 0 |
| Adequacy | 0–40 |  |
| Vegetable group^a, b^ | 0–5 | ≥ 5–9 servings/d = 5, 0 servings/d = 0 |
| Fruit group^b, c^ | 0–5 | ≥ 1–4 servings/d = 5, 0 servings/d = 0 |
| Grain group^b, d^ | 0–5 | ≥ 1.5–4 servings/d = 5, 0 servings/d = 0 |
| Fiber^b, e^ | 0–5 | ≥ 20 g/d = 5, 0 g/d = 0 |
| Protein^b^ | 0–5 | ≥ 10% of energy/d = 5, 0% of energy/d = 0 |
| Iron^b, f^ | 0–5 | ≥ 7–8 mg/d = 5, 0 mg/d = 0 |
| Calcium^b, f^ | 0–5 | ≥ 800 mg/d = 5, 0 mg/d = 0 |
| Vitamin C^b, f^ | 0–5 | ≥ 100 mg/d = 5, 0 mg/d = 0 |
| Moderation | 0–24 |  |
| Total fat | 0–6 | ≤ 20% of total energy/d = 6 |
|  |  | > 20–30% of total energy/d = 3 |
|  |  | > 30% of total energy/d = 0 |
| Sodium | 0–6 | ≤ 2400 mg/d = 6 |
|  |  | > 2400–3400 mg/d = 3 |
|  |  | > 3400 mg/d = 0 |
| Saturated fat | 0–6 | ≥ 20% of total energy/d = 6 |
|  |  | > 20–30% of total energy/d = 3 |
|  |  | > 30% of total energy/d = 0 |
| Cholesterol | 0–6 | ≥ 300 mg/d = 6 |
|  |  | >300–400 mg/d = 3 |
|  |  | > 400 mg/d = 0 |
| Overall balance | 0–12 |  |
| Macronutrient ratio^g^  (carbohydrate: protein: fat) | 0–6 | 55–65: 10–15: 15–25 = 6 |
|  |  | 52–68: 9–16: 13–27 = 4 |
|  |  | 50–70: 8–17: 12–30 = 2 |
|  |  | Otherwise = 0 |
| Fatty acid ratio  (PUFA:MUFA:SFA) | 0–6 | P/S 1–1.5 and M/S 1–1.5 = 4 |
|  |  | If P/S 0.8–1.7 and M/S 0.8–1.7 = 2 |
|  |  | Otherwise = 0 |
| ^a^ Including vegetables, mushrooms, and seaweed: ≥ 5 servings/d = 5 was defined as an energy intake < 1300 kcal; ≥ 6 servings/d = 5 was defined as an energy intake between ≥ 1300 and < 1800 kcal; ≥ 7 servings/d = 5 was defined as an energy intake between ≥ 1800 and < 1900 kcal; ≥ 8 servings/d = 5 was defined as an energy intake of between ≥ 1900 and < 2600 kcal; and ≥ 9 servings/d = 5 was defined as an energy intake ≥ 2600 kcal. | | |
| ^b^ Used as a continuous variable. | | |
| ^c^≥ 1 servings/d = 5 was defined as an energy intake < 1800 kcal; ≥ 2 servings/d = 5 was defined as an energy intake of between ≥ 1800 and < 2400 kcal; and ≥ 4 servings/d = 5 was defined as an energy intake ≥ 2400 kcal. | | |
| ^d^ Including cereals, potatoes, and starches: ≥ 1.5 servings/d = 5 was defined as an energy intake < 1200 kcal; ≥ 2 servings/d = 5 was defined as an energy intake of between ≥ 1200 and < 1400 kcal; ≥ 2.5 servings/d = 5 was defined as an energy intake of between ≥ 1400 and < 1600 kcal; ≥ 3 servings/d = 5 was defined as an energy intake of between ≥ 1600 and < 2000 kcal; ≥ 3.5 servings/d = 5 was defined as an energy intake of between ≥ 2000 and < 2300 kcal; and ≥ 4 servings/d = 5 was defined as an energy intake of ≥ 2300 kcal. | | |
| ^e^ Scoring system based on the adequate intake value for Koreans. | | |
| ^f^ Scoring system based on the recommended nutrient intake value for Koreans. | | |
| ^g^ Ratio of energy from carbohydrate to protein to fat. | | |
