# Supplementary material for: Poor diet quality is associated with self-reported knee pain in community-dwelling women aged 50 years and older
Source: PLoS One. 2021 Feb 16;16(2):e0245630. doi: 10.1371/journal.pone.0245630 (PMC7886155; doi:10.1371/journal.pone.0245630)
Supplement: S2 Table — (DOCX) [file pone.0245630.s002.docx]

**S2 Table.** **Intake of micronutrients according to the DQI-I score quartiles**

| Micronutrients | DQI-I score | | | | P-value^a^ |
| --- | --- | --- | --- | --- | --- |
|  | 1^st^ Quartile | 2^nd^ Quartile | 3^rd^ Quartile | 4^th^ Quartile |  |
| Calcium (mg/d) | 312.6 ± 8.9 | 396.8 ± 9.1 | 454.7 ± 9.3 | 571.0 ± 10.6 | < 0.01 |
| Phosphorous (mg/d) | 693.3 ± 15.2 | 850.0 ± 14.7 | 950.1 ± 14.2 | 1149.6 ± 15.3 | < 0.01 |
| Iron (mg/d) | 11.5 ± 0.3 | 15.0 ± 0.3 | 17.4 ± 0.6 | 20.0 ± 0.6 | < 0.01 |
| Potassium (mg/d) | 1933.4 ± 46.2 | 2633.5 ± 50.5 | 3103.0 ± 52.2 | 3835.2 ± 66.2 | < 0.01 |
| Vitamin A (µgRE/d) | 503.4 ± 30.6 | 659.6 ± 34.3 | 755.9 ± 40.3 | 849.4 ± 46.4 | < 0.01 |
| Thiamine (mg/d) | 1.4 ± 3.2E^-2^ | 1.6 ± 2.6E^-2^ | 1.8 ± 2.7E^-2^ | 2.1 ± 2.8E^-2^ | < 0.01 |
| Riboflavin (mg/d) | 0.9 ± 2.5E^-2^ | 1.0 ± 2.3E^-2^ | 1.1 ± 2.3E^-2^ | 1.3 ± 2.3E^-2^ | < 0.01 |
| Niacin (mg/d) | 10.1 ± 0.3 | 12.3 ± 0.3 | 13.8 ± 0.3 | 16.6 ± 0.3 | < 0.01 |
| Vitamin C (mg/d) | 50.0 ± 2.9 | 100.7 ± 5.0 | 145.8 ± 5.7 | 181.4 ± 6.4 | < 0.01 |

^a^Using a weighted analysis of covariance (ANCOVA) adjusted for age and body mass index. It was performed using PROC SURVEYREG.
